# Drought-tolerant *Sphingobacterium changzhouense* Alv associated with *Aloe vera* mediates drought tolerance in maize (*Zea mays*)

**DOI:** 10.1007/s11274-022-03441-y

**Published:** 2022-10-28

**Authors:** Noura Sh. A. Hagaggi, Usama M. Abdul-Raouf

**Affiliations:** Botany Department, Faculty of Science, Aswan University, Aswan, 81528 Egypt

**Keywords:** *Aloe vera*, *Sphingobacterium changzhouense*, Maize, Drought, Stress

## Abstract

Drought severity and duration are expected to increase as a result of ongoing global climate change. Therefore, finding solutions to help plants to deal with drought stress and to improve growth in the face of limited water resources is critical. In this study, a drought tolerant- plant growth promoting endophytic bacterium was isolated from *Aloe vera* roots. It was identified as *Sphingobacterium changzhouense* based on 16S rRNA gene sequencing and was deposited into NCBI database with accession number (ON944028). The effect of *S. changzhouense* inoculation on maize growth under drought stress was investigated. The results revealed that inoculation significantly (*p* ≤ 0.05) enhanced root and shoot elongation by 205 and 176.19% respectively. Photosynthesis rate, stomatal conductance and water use efficiency were improved in inoculated plants. interestingly, inoculation resulted in significant increase in total chlorophyll, total carbohydrates, proline, total proteins, total phenolics and total flavonoids by 64, 31.5, 25.1, 75.07, 83.7 and 65.4% respectively. Total antioxidant capacity of inoculated plants (51.2 mg/g FW) was higher than that of non-inoculated plants (11.87 mg/g FW), which was found to be positively correlated to the levels of phenolics and flavonoids. Our finding suggests that *S. changzhouense* could be used to improve crop growth and assist plants to resist drought stress in arid agricultural lands.

## Introduction

Crop growth and production in many parts of the world have been negatively affected by global climate change, which has resulted in an increase in drought and extreme temperature periods (Grinnan et al. 2013). Drought stress is one of the most critical issues impacting plant growth, development, and production (Mir et al. 2012; Prasanna 2012). It has a variety of effects on plants, including reducing water and nutrient uptake, restricting photosynthesis, and disrupting plasma membranes (Ge et al. 2012). Drought-affected arable lands on the planet have doubled in recent years (Isendahl and Schmidt 2006).

Cereal crops have a significant role in human and animal food systems and significantly contribute to global food security (Zmaic et al. 2007). Maize (*Zea mays* L.) is an important multipurpose economic cereal crop of the world (Harris et al. 2007). It ranks second after wheat and is equivalent to rice. It participates in the human diet, animal feed, fodder, and bioenergy production (Nyakurwa et al. 2017). Maize plants are highly oversensitive to drought conditions that hamper the growth and yield (Lobell et al. 2011; Zafar-ul-Hye et al. 2014).

Until now, a technique aimed at creating drought-tolerant cultivars has been employed to alleviate the detrimental impacts of drought stress on crops, but it comes with its own set of obstacles, including time consuming and labor cost (Ashraf 2010; Eisenstein 2013). Therefore, the development of microbial-based approaches to mitigate drought stress is of an interest. Currently, plant-associated microorganisms have received attention for enhancing crop stress resistance (Marulanda et al., 2009; Yang et al. 2009). Many beneficial microorganisms have capacity to produce a wide range of enzymes and metabolites help plants to tolerate drought stress (Kim et al. 2009; Pineda et al. 2013; Chauhan et al. 2015). Among these microorganisms are endophytic bacteria that inhabit the internal tissues of plants without causing any adverse effect on the host plant (Ryan et al. 2008). It was reported that the inoculation of plants with endophytic bacteria resulted in water and nutrient uptake enhancement, transpiration regulation, phytohormones induction, antioxidative and photosynthetic improvement, thereby ensuring plant survival under stress conditions (Marulanda et al. 2010; Marasco et al. 2012).

*Aloe vera* is perennial, succulent plant belongs to the family Asphodelaceae (Liliaceae). It grows in hot, dry climates and has various medicinal benefits (Surjushe et al. 2008). It was selected for this study because it is a drought tolerant plant, and whereas endophytes can confer habitat-specific stress tolerance to their hosts (Rodriguez et al. 2008), we expected that bacteria associated with *aloe vera* may have a role in alleviating drought stress in plants. Although some *Sphingobacterium* spp., such as *Sphingobacterium pakistanensis* and *Sphingobacterium* sp. BHU-AV3, have been shown to promote plant development under various stress conditions, no studies have been performed on *Sphingobacterium changzhouense* (Ahmed et al. 2014; Vaishnav et al. 2020). As a result, this is the first study to investigate the role of *Sphingobacterium changzhouense* in stimulating plant growth under drought stress. This study aims to evaluate the effect of inoculation with the endophytic *Sphingobacterium changzhouense* isolated from *Aloe vera* on maize growth under drought stress.

## Materials and methods

### Isolation and identification of endophytic bacteria

*Aloe vera* root samples were collected from Aswan University, Egypt (39.59°N 32.82°E). Samples were immediately surface sterilized using 5% sodium hypochlorite (1 min), then 70% ethanol (1 min) and washed three times with sterilized distilled water. Samples were homogenized in sterilized saline solution and filtered. 1 mL of the filtrate was spread into trypticase soy and nutrient agar plates. Plates were incubated at 37 °C with daily observation for 72 h. The selected isolate was coded as Alv and sent to Korea Solgent Lab for molecular identification by 16S rRNA gene sequencing. The obtained sequence was subjected to blast analysis using NCBI website (https://www.ncbi.nlm.nih.gov/) and the percent of similarity with other reference sequences in NCBI database was determined. The present sequence was introduced to NCBI and an access number has been obtained. The MEGA X software was used for the construction of a phylogenetic tree (Kumar et al. 2018).

### Assay of drought tolerance of the isolate

The drought tolerance by the isolate was determined according to Saad and Abo-Koura (2018). The isolate was grown in nutrient broth supplemented with different concentrations of polyethylene glycol 6000 i.e., 0, 10, 20 and 30% at 37  °C with shaking (150 rpm) for 72 h. Then, the optical density was measured by spectrophotometer at 600 nm.

### Assay of temperature tolerance of the isolate

To determine the effect of temperature on the growth of the isolate, 250-mL flasks contained 50 mL of sterilized nutrient broth were inoculated with 100 µL of standard inoculum (10^8^ CFU/mL). Flasks were incubated at different temperatures i.e., 40, 45, 50 and 55 °C for 72 h. The optical density was measured by spectrophotometer at 600 nm.

### Determination of plant growth-promoting activities of the isolate

#### Indole acetic acid (IAA) production

The bacterial strain was grown in conical flasks contained 100 mL nutrient broth supplemented with 1 g of L-tryptophan (Sigma-Aldrich). Standard inoculum containing about 10^8^ CFU/mL was prepared from 3-day-old bacterial culture using McFarland method (McFarland, 1907). Flasks were inoculated with 1 mL of the inoculum and incubated with shaking (150 rpm) for 72 h at 37 °C. Salkowski method was used to evaluate IAA in the supernatant after centrifuging the culture (Glickmann and Dessaux 1995).

#### Gibberellic acid (GA3) production

To evaluate the gibberellic acid production by the strain, the method described by Berríos et al. (2004) was followed. Briefly, in 10 mL volumetric flask, 1 mL of bacterial supernatant was vigorously mixed with 1 mL absolute ethanol and 8 mL HCl (3.75 M). Absorbance of the mixture was recorded at 254 nm for 2 min in 20 s interval. The gibberellic acid standard curve was used to calculate the gibberellic acid content.

#### Exopolysaccharide (EPS) production

The method of Naseem and Bano (2014) was followed to estimate the production of EPS by the isolate. The isolate was cultured in mineral salt medium contained (%): K_2_HPO_4_, 12.6, KH_2_PO_4_, 18.2, NH_4_NO_3_, 10, MgSO_4_.7H_2_O, 1, MnSO_4_, 0.6, CaCl_2_.2H_2_O, 1, FeSO_4_.2H_2_O, 0.06, sodium molybdate, 1, NaCl, 1.5 and glucose, 0.2). Culture was incubated in a shaker incubator (150 rpm) for 7 days at 37 °C. Then, culture was centrifuged, and supernatant was mixed with two volumes of cold absolute ethanol. The precipitate was collected, washed, dried and weighted.

#### Phosphate solubilization

The strain was spot inoculated on Pikovskaya’s agar plates. Plates were incubated at 37 °C for 7 days. Diameter of clear halo zones appeared around the growth was measured (Karpagam and Nagalakshmi 2014).

#### Effect of bacterial inoculation on maize growth under drought stress

The effect of isolate Alv inoculation on the growth of maize under drought stress was investigated. Maize grains (TWC 321) were obtained from the Agricultural Research Center, Egypt. Grains were surface sterilized with sodium hypochlorite (5%) for 3 min, then washed three times with sterilized distilled water. Sterilized grains were soaked in 50 mL bacterial suspension (10^8^ CFU/mL) of isolate Alv and sterilized distilled water (Control) for 3 h. Inoculated grains were sown in plastic pots containing autoclaved soil mixture (2:1, v/v) of clay and sand supplemented with bacterial inoculum (100 mL/Kg soil). Control pots received sterilized distilled water. Pots were divided into three groups as follow: the first group (control under normal irrigation) is non-inoculated subjected to water regime 90% field capacity, while the second group (control under drought stress) is non-inoculated subjected to water regime 35% field capacity, and the third group (treatment under drought stress) is inoculated with isolate Alv and subjected to water regime 35% field capacity. Pots were kept under normal climatic conditions. After two months of sowing, plants were collected for evaluation of growth and physiological parameters as well as biochemical constituents. The experiment was repeated twice with five replicates.

### Measurement of growth parameters

Root length, shoot length as well as fresh and dry biomasses were measured for randomly selected plants.

### Measurement of gas-exchange and photosynthesis parameters

The net photosynthesis rate (Pn), transpiration rate (E), leaf stomatal conductance (C) and leaf water-use efficiency (WUE) were measured for randomly selected fully expanded healthy leaves. Measurements were carried out in controlled leaf chamber using infrared gas analyzer (IRGA, CI 340) photosynthesis system (CID Bio-Science, Inc.). Levels of photosynthetically active radiation (PAR) and incoming air CO_2_ were set at 1500 μmol m^−2^ s ^−1^ and 360 ppm respectively. Relative humidity and temperature in the leaf chamber were 50% and 26 ± 0.1 °C. Data was recorded between 12:57 and 2:30 PM.

### Measurement of biochemical constituents

#### Total chlorophyll

Chlorophyll content was measured from the expanded leaves of maize plants. About 1 g leaves was homogenized and extracted with 10 mL of 80% acetone (Arnon 1949). The contents of chlorophylls *a* and *b* in the filtrates were estimated by reading the absorbance at 645 and 663 nm respectively using UV–visible spectrophotometer (UVmini-1240, Shimadzu Corporation, Japan). The total chlorophyll was calculated according to the following formula: Total chlorophyll (mg/g FW) = 20.2 A_645_ + 8.02 A_663_ (Porra et al. 1989).

#### Total carbohydrates

Morris (1948) method was used to measure the content of carbohydrates in the seedlings. Briefly, 1 g of plant materials were hydrolyzed for 2 h at 100 °C using HCl (4 N). The hydrolysates were cooled and filtered. 9 mL of 2% (w/v) anthrone reagent prepared in concentrated H_2_SO_4_ was added to 1 mL of sample filtrates. Then, the reaction mixtures were heated for 7 min and cooled. The absorbance was measured at 630 nm using UV–visible spectrophotometer (model UVmini-1240, Shimadzu Corporation, Japan). The content of carbohydrates (mg/ g fresh weight) was calculated using a standard curve of glucose.

#### Proline content

The method of Bates et al. (1973) was followed to estimate proline content. 1 g of plant tissue was homogenized in 10 mL of 3% sulphosalicylic acid and centrifuged. 2 mL of acid ninhydrin reagent (2.5 g ninhydrin dissolved in 40 mL of orthophosphoric acid (6 M) and 60 mL of glacial acetic acid) was mixed with 2 mL of the supernatant and 2 mL of glacial acetic acid. The reaction mixture was heated at 100 °C for 1 h. After cooling, 4 mL of toluene was added. Absorbance was measured at 520 nm. Proline content was quantified using proline standard curve.

#### Total proteins

Total proteins content of seedlings was estimated using Lowry assay (Lowry et al. 1951). Reagent A was prepared by dissolving 2 g of Na_2_CO_3_ in 100 mL NaOH (0.1 N) and reagent B was prepared by dissolving 0.5 g of CuSO_4_⋅5H_2_O in 100 mL sodium- potassium tartarate (1%). Reagent C was prepared by mixing 50 mL of reagent A with 1 mL of reagent B. 1 g of plant material was mixed with 5 mL of reagent C and was incubated at room temperature for 15 min. Then, 0.5 mL of Folin–Ciocalteau reagent was added to the mixture and was left for 30 min. The absorbance against the blank was measured at 700 nm. Bovine Serum Albumin (Sigma-Aldrich) was used to construct the calibration curve. The content of protein was expressed as mg/g fresh weight.

#### Total phenolics, total flavonoids and total antioxidant capacity (TAC)

One gram of fresh plant tissue was macerated in 40 mL of 80% methanol, vortexed and placed in water bath at 60 °C for 1 h. The extracts were centrifuged and filtered. The obtained filtrates were used for estimation of total phenolics, total flavonoids and total antioxidant capacity. Total phenolics content was evaluated by Folin-Ciocalteu reagent method according to Singleton et al. (1999) and expressed as mg gallic acid equivalents per gram of fresh weight. The content of total flavonoids was estimated using the aluminum chloride colorimetric assay and was expressed as mg quercetin equivalents per gram of fresh weight (Zhishen et al. 1999). Phosphomolybdenum assay was used to estimate the total antioxidant capacity and was expressed as mg ascorbic acid equivalents per gram of fresh weight using ascorbic acid as a reference (Prieto et al. 1999).

### Statistical analysis

Data obtained were subjected to one-way analysis of variance (ANOVA) using Minitab 18 software. Values are means ± standard errors of five biological replicates (n = 5) obtained from two independent experiments. The significant differences between means were computed by Tukey's HSD test at p ≤ 0.05.

## Results

### Isolation and identification

**Fig. 1:**
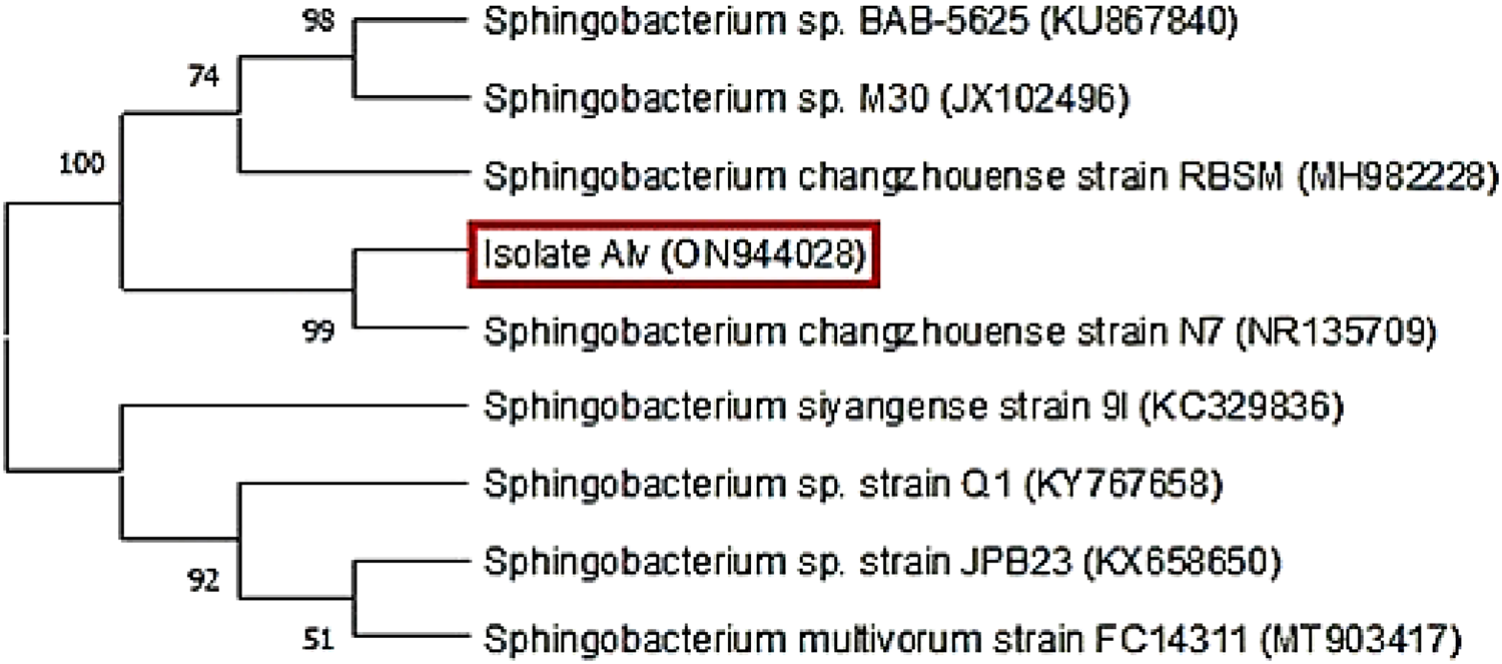
Neighbor-joining phylogenetic tree based on 16S rRNA gene sequencing showing the relationship between isolate Alv and closely related bacteria derived from NCBI GenBank

The comparative sequence analysis of isolate Alv with NCBI GenBank database using BLAST tool showed that isolate Alv belongs to genus *Sphingobacterium* and exhibited the highest similarity percent with *Sphingobacterium changzhouense* strain N7 (NR135709) (Fig. 1). The 16S rRNA gene sequence of the present isolate was deposited to NCBI GenBank under the accession number (ON944028).

### Drought tolerance of the isolate

**Fig. 2:**
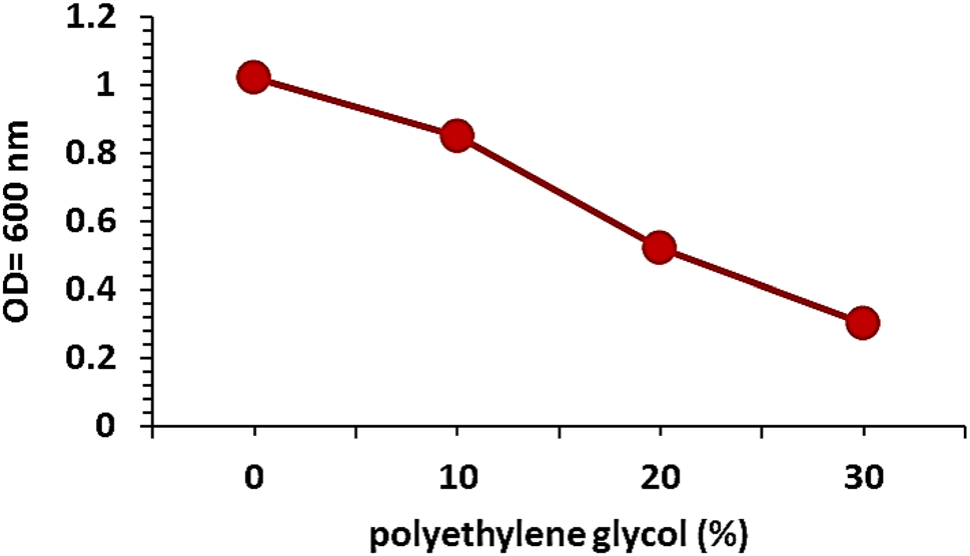
Drought tolerance of isolate Alv

Isolate Alv was screened for its drought tolerance by growing it at different concentrations of polyethylene glycol. It was grown at polyethylene glycol concentrations ranged from 0 to 30% (Fig. 2).

### Temperature tolerance of the isolate

**Fig. 3:**
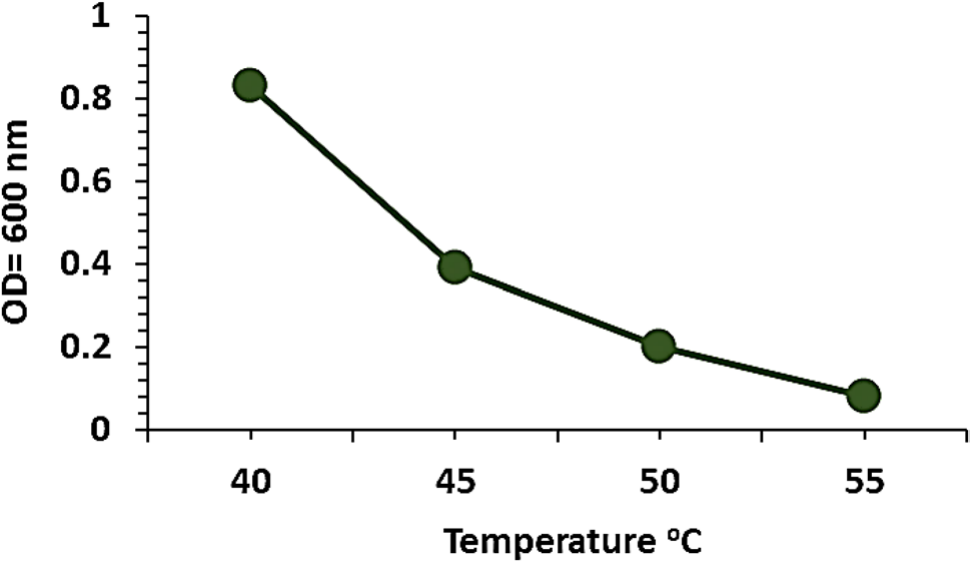
Temperature tolerance of isolate Alv

The isolate was grown at different temperatures to evaluate its temperature tolerance. The results showed that it could tolerate up to 45 °C, above this the growth was declined (Fig. 3).

### Plant growth-promoting activities of the isolate

The present isolate was screened for plant growth promoting activities including indole acetic acid, gibberellic acid, and exopolysaccharide production as well as phosphate solubilization. Significant activities were noted (Table 1).

**Table 1:** Plant growth-promoting activities of isolate Alv

Activities	Result
Indole acetic acid (µg/mL)	2116 ± 0.57
Gibberellic acid (µg/mL)	1221 ± 1.15
Exopolysaccharide (mg/mL)	1.93 ± 0.04
Phosphate solubilization (mm)	28 ± 1.41

Values are means ± standard errors of five biological replicates (n = 5)

### Effect of bacterial inoculation on maize growth under drought stress

The effect of the inoculation with isolate Alv on the growth and physiological parameters as well as biochemical constituents of maize grown under drought stress was evaluated.

### Growth parameters

Table 2 shows the growth characteristics for non-inoculated and inoculated plants, including root and shoot length and fresh and dry biomass. Drought stress was found to be detrimental to maize growth, resulting in significant reductions in all assessed growth parameters. Inoculation with isolate Alv, on the other hand, had a statistically significant favorable effect on maize growth as compared to non-inoculated plants.

**Table 2:** Effect of Alv-inoculation on growth parameters of maize under drought stress

Growth parameters	Non-inoculated + 90% FC	Non-inoculated + 35% FC	Inoculated + 35% FC
Root length (mm)	45 ± 0.57^a^	20 ± 0.15^b^	61 ± 0.57^c^
Shoot length (mm)	120 ± 1.52^a^	63 ± 0.14^b^	174 ± 0.57^c^
Fresh biomass (mg)	184 ± 0.57^a^	80 ± 0.57^b^	231 ± 0.15^c^
Dry biomass (mg)	59 ± 1.52^a^	35 ± 1.52^b^	74 ± 1.52^c^

Values are means ± standard errors of five biological replicates (n = 5). Different letter alphabets (a, b, c) refer to significant differences between plants at *p* ≤ 0.05 according to Tukey's HSD test analysis. FC = Field capacity

### Gas-exchange and photosynthesis parameters

**Fig. 4:**
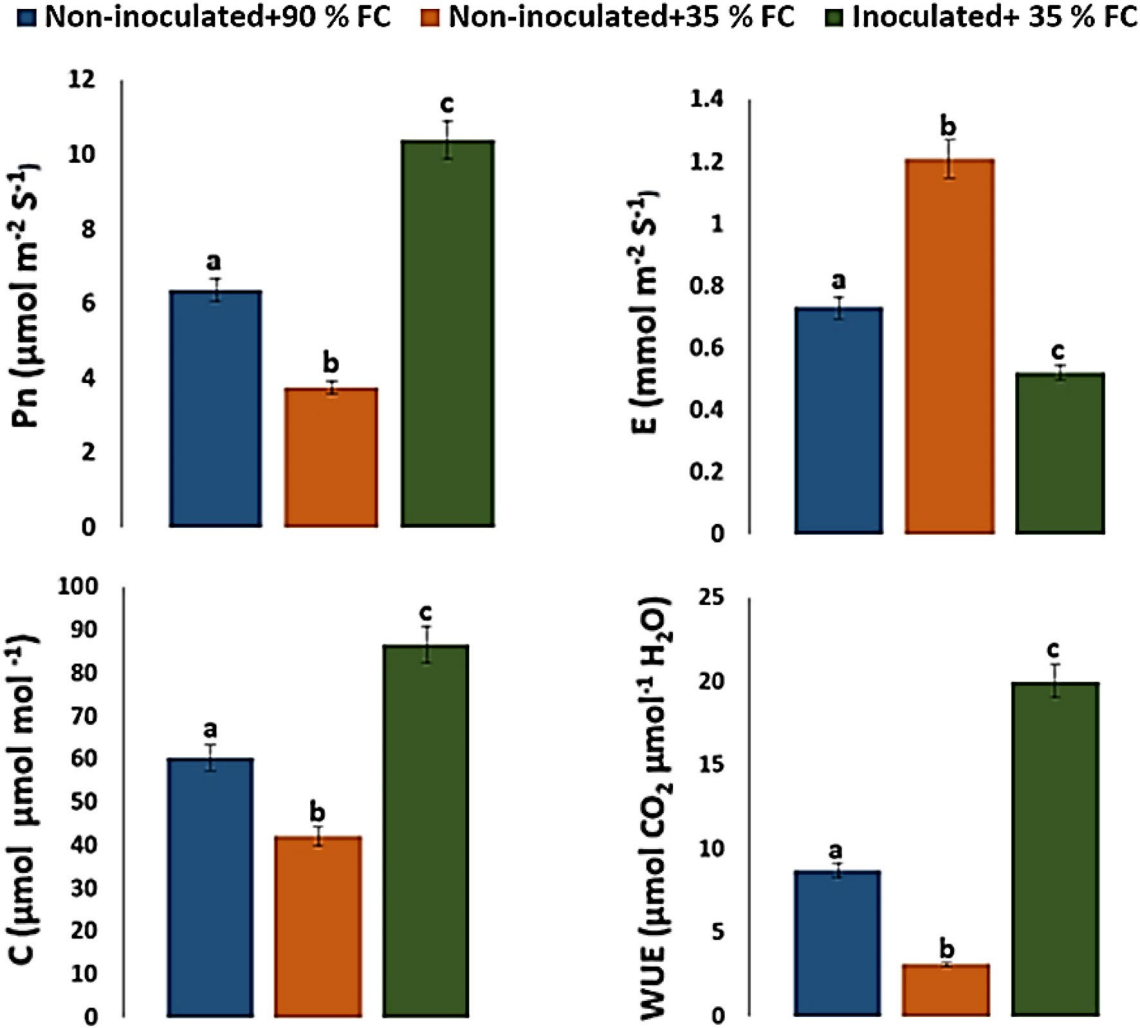
Effect of Alv-inoculation on photosynthesis rate (Pn), transpiration rate (E), stomatal conductance (C) and water-use efficiency (WUE) in maize plants under drought stress. a, b, c indicates significant differences (*p* ≤ 0.05) between inoculated and non-inoculated plants. *FC* field capacity

Drought stress significantly (*p* ≤ 0.05) decreased the net photosynthesis rate, stomatal conductance, and water-use efficiency in non-inoculated plants. Significantly, inoculation with isolate Alv enhanced gas-exchange and photosynthesis in maize plants under drought stress (Fig. 4). Photosynthesis rate, stomatal conductance, and water use efficiency in inoculated plants increased by 104.7, 73.8 and 194.2% compared with non-inoculated plants respectively. On the other hand, inoculated plants exhibited significant decrease in transpiration rate by 57% compared with non-inoculated plants under drought stress.

### Biochemical constituents

**Fig. 5:**
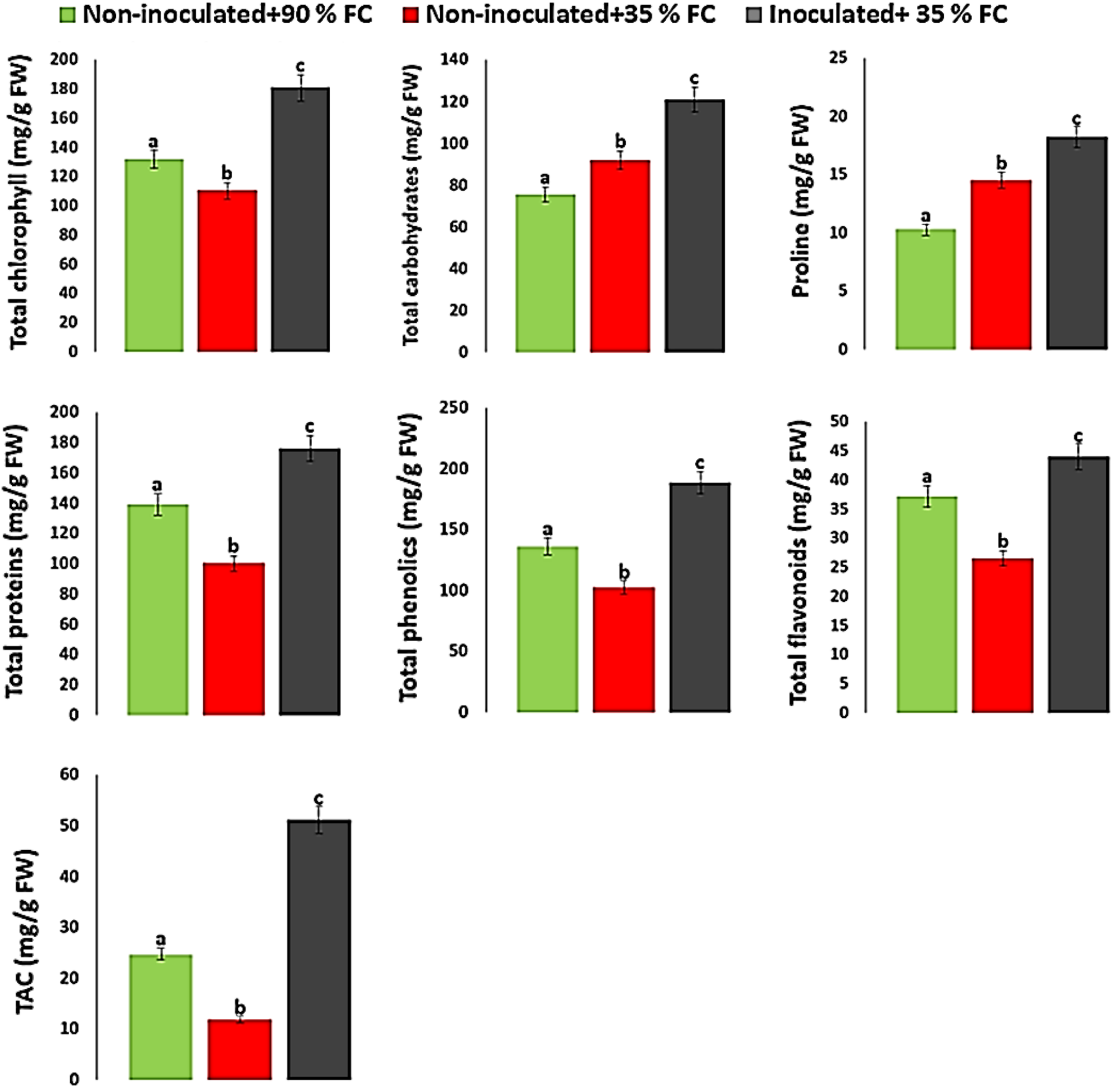
Differences in total chlorophyll, total carbohydrates, proline content, total proteins, total phenolics, total flavonoids and total antioxidant capacity (TAC) (mg/g FW) between inoculated and non-inoculated plants. a, b, c indicates significant differences (p ≤ 0.05) between inoculated and non-inoculated plants. *FC* field capacity

Differences in biochemical constituents between inoculated and non-inoculated maize plants under drought stress were represented by (Fig. 5). Under stress condition, the leaf chlorophyll content of plants was decreased by 16.26% as compared with their respective control (under normal irrigation). Inoculation with isolate Alv increased chlorophyll content in stressed plants by 64% compared with stressed non-inoculated plants. Inoculated maize plants showed significant increase (*p* ≤ 0.05) in total carbohydrates under drought stress compared with non-inoculated plants (Fig. 5). Moreover, the contents of proline, proteins, phenolics and flavonoids were increased under drought stress in inoculated plants by quantum of 25.1, 75.07, 83.7 and 65.4% respectively over the non-inoculated. Under drought stress, it was noted that the antioxidant capacity of the inoculated plants (51.2 mg/g FW) was higher than that of non-inoculated plants (11.87 mg/g FW), which was positively correlated with the contents of phenolics and flavonoids.

## Discussion

Drought is a major challenge to crop development and productivity in many places of the world (Vinocur and Altman 2005; Naveed et al. 2014). Drought is expected to cause major plant development problems for crops on more than half of the world's arable lands by 2050 (Vinocur and Altman 2005). Therefore, crop drought tolerance enhancement is regarded as the most pressing concern. Consequently, the effect of *Sphingobacterium changzhouense* Alv inoculation on maize growth under drought stress was investigated in this study.

*S. changzhouense* Alv was isolated from the roots of drought-adapted plant *Aloe vera*. It exhibited significant drought and temperature tolerance (Figs. 2, 3). This may be because the bacterial cell can protect its structures and organelles under heat- drought conditions through accumulation of compatible solutes such as proline, glycine betaine and trehalose as well as exopolysaccharides that increase thermostability of enzymes, inhibits proteins thermal denaturation, and maintain membrane integrity (Welsh 2000; Conlin and Nelson 2007; Bérard et al. 2015).

**Fig. 6:**
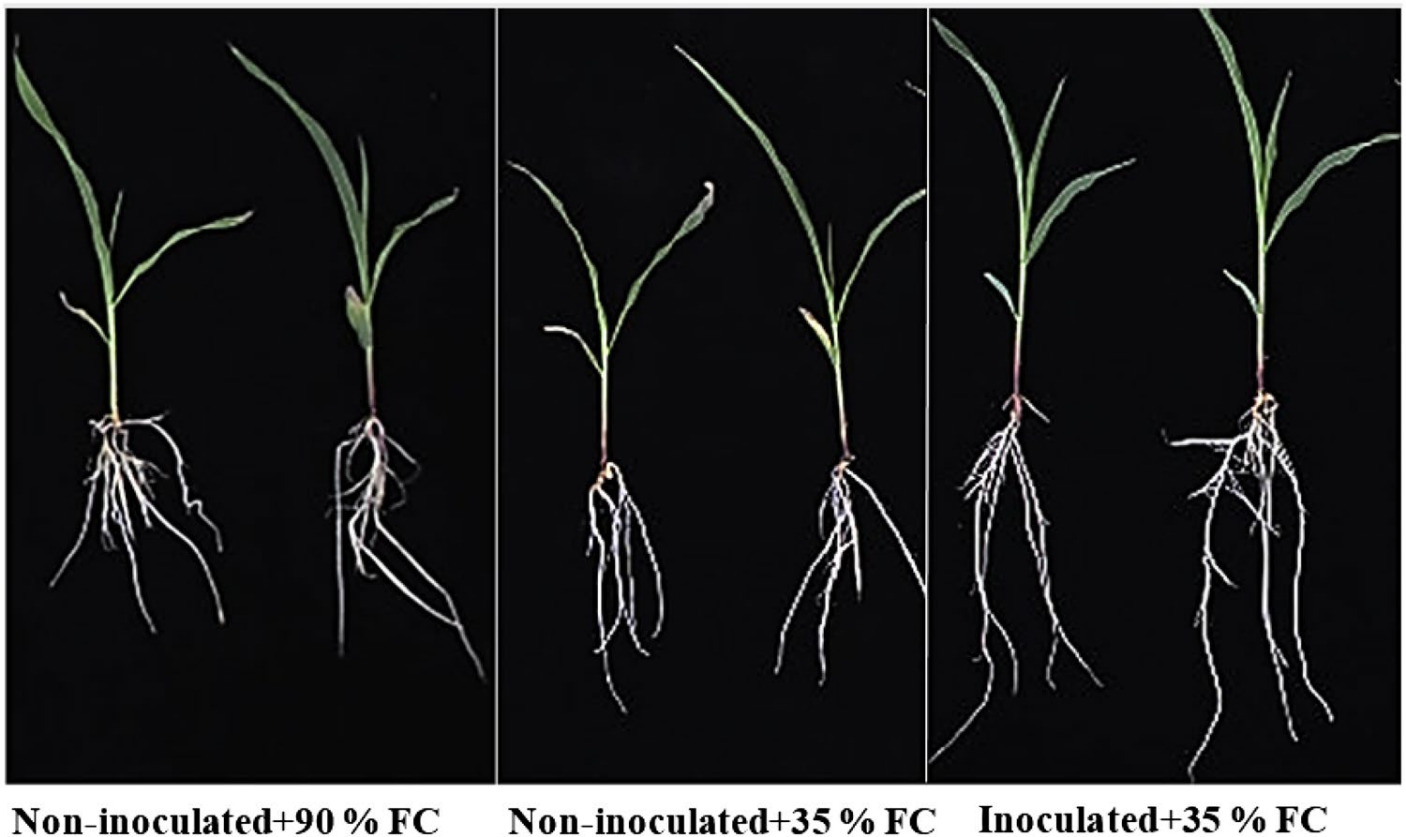
Effect of Alv-inoculation on maize growth under drought stress

Interestingly, the whole growth of maize under drought stress was improved upon inoculation with isolate Alv compared to drought stressed non-inoculated plants (Fig. 6). Drought stressed inoculated maize plants showed an increase in root and shoot lengths by 205 and 176.19% compared to drought stressed non-inoculated plants (Table 2). This may be because isolate Alv produces phytohormones like indole acetic acid and gibberellic acid (Table) that stimulate cell division as well as elongation of roots and stems (Glick 1995). Improvement of root and shoot systems enables plants undergoing drought to increase water and nutrient uptake as well as photosynthesis efficiency and consequently enhances plant growth (Timmusk et al. 2014). The improvement of root and shoot growth of maize via bacterial inoculation was previously reported (Vardharajula et al. 2011; Naveed et al. 2014).

Photosynthesis is an important physico-chemical process that directly effects on plant growth and biomass production (Yang et al. 2014). Drought stress alters physiological processes, it disrupts photosynthetic pigments, reduces gas exchange and stomatal function, and consequently causes reductions in net photosynthesis (Keyvan 2010). In the present study, photosynthetic parameters in non-inoculated plants were strongly affected by drought stress (Fig. 4). On the other hand, the inoculation with isolate Alv significantly (*p* ≤ 0.05) increased gas exchange and photosynthetic parameters. Interestingly, inoculation increased the net photosynthesis rate under drought stress by 104.7% over the non-inoculated plants (Fig. 4). This may be because inoculation enhances stomatal conductance that regulates gas exchange and subsequently enhances photosynthesis (Kusumi et al. 2012).

Water use efficiency is one of the most crucial factors limiting crop production worldwide and its improvement is of major concern with drought problems (Waraich et al. 2011). In the present study, water use efficiency was significantly (*p* ≤ 0.05) increased in drought-stressed inoculated plants compared with drought-stressed non-inoculated plants (Fig. 4). High water use efficiency combined with stomatal conductance showed that isolate Alv inoculation can be beneficial for water transportation through plants and can help plants keep their stomata open. Therefore, the response may be an important mechanism for maize plants to adapt to drought stress. The obtained results are like those obtained in previous studies where enhancement of water use efficiency and stomatal conductance upon inoculation of maize with *Pseudomonas* spp. was reported (Sandhya et al. 2010).

The content of photosynthetic pigments is an important physiological indicator for drought tolerance (Pour-Aboughadareh et al. 2020). In this study, the total chlorophyll content was significantly increased by 64% in plants upon inoculation with isolate Alv (Fig. 5).

In the present study, it was noted that the total carbohydrates content was significantly increased in inoculated plants compared with non-inoculated plants during drought stress (Fig. 5). The accumulation of carbohydrates is part of a wider mechanism for plant surviving during drought stress that plays a key role in the regulation of carbon metabolism (Praxedes et al. 2005). Increases in carbohydrates content attributable to bacterial inoculation under drought stress conditions was previously documented (Heidari et al. 2011; Kalita et al. 2015; Omara et al. 2017).

Proline is one of the most crucial compatible solutes that accumulates in drought-stressed plants (Farooq et al. 2008). It contributes to stabilizing proteins and membranes as well as scavenging free radicals (Ashraf and Foolad 2007; Hayat et al. 2012). In this study, the proline level accumulated by inoculated maize plants under drought stress was higher by 76.89% than those accumulated by non-inoculated plants. Increase in proline levels upon bacterial inoculation has been demonstrated in maize under drought stress (Sandhya et al. 2010; Vardharajula et al. 2011; Naseem and Bano 2014).

The total proteins significantly (*p* ≤ 0.05) increased in inoculated plants compared with non-inoculated plants under drought stress (Fig. 5). Increasing in protein content under drought stress was previously reported (Qaseem et al. 2019). On the other hand, the total phenolics and total flavonoids showed significant increases in the inoculated plants comparing to non-inoculated plants (Fig. 5). These compounds were found to prevent tissues from oxidative damage and enable plants to tolerate stresses (Pazoki 2015; Ilangumaran and Smith 2017; Nawaz and Bano 2020). Our results agreed with those obtained by Jha (2017) who reported that phenolics and flavonoids were enhanced in PGPB-inoculated maize under normal and stress conditions. Furthermore, the total antioxidant capacity was significantly (*p* ≤ 0.05) increased in inoculated plants compared with the non-inoculated plants under drought stress (Fig. 5). In this study, positive correlation was noted between total antioxidant capacity and phenolics as well as flavonoids contents. The presence of positive correlation between phenolics, flavonoids and antioxidant activities has been previously reported (Ghasemzadeh et al. 2012; Baharfar et al. 2015). Improvement of antioxidant capacity mediated by bacterial inoculation under drought stress was previously documented (Erdogan et al. 2016; Nawaz and Bano 2020).

## Conclusion

In the current study, the endophyte *Sphingobacterium changzhouense* Alv was isolated from *Aloe vera*. It showed multiple plant growth-promoting activities as well as drought and temperature tolerance. The results of this study provide evidence that inoculation with *S. changzhouense* can enhance drought tolerance of maize through improving plant growth and physio-biochemical status. So, the application of endophyte inoculation approach should be encouraged in dryland farming in order to improve crop growth and drought resistance.
